# Voxel-wise lesion mapping of restless legs syndrome in multiple sclerosis

**DOI:** 10.1007/s10072-022-06103-x

**Published:** 2022-05-05

**Authors:** Kilian Fröhlich, Michael Knott, Stefan Hock, Arnd Dörfler, Frank Seifert, Klemens Winder

**Affiliations:** 1 Department of Neurology, University Hospital Erlangen, Friedrich-Alexander-University Erlangen–Nürnberg (FAU), Schwabachanlage 6, 91054 Erlangen, Germany; 2 Department of Neuroradiology, University Hospital Erlangen, Friedrich-Alexander-University Erlangen–Nürnberg (FAU), Erlangen, Germany

**Keywords:** Multiple sclerosis, Voxel-based lesion symptom mapping, Restless legs syndrome

## Abstract

**Objective:**

Restless legs syndrome (RLS) is known to be associated with multiple sclerosis (MS) and may be caused by MS lesions in specific cerebral brain regions. Applying a voxel-wise lesion analysis, we tried to identify the contribution of cerebral MS lesions to RLS.

**Methods:**

In this retrospective study, we established a cohort of people with MS with documented RLS and controls of people with MS without RLS matched disease severity. Diagnosis of MS and RLS was based on the current guidelines. The MS lesions were analyzed on T2-weighted magnetic resonance imaging scans (1.5 or 3 T). After manual delineation, lesion maps were converted into stereotaxic space. We generated a lesion overlap and performed a Liebermeister test with 4000 permutations to compare the absence or presence of RLS voxel-wise between patients with and without lesions in a given voxel.

**Results:**

Forty of the patients with RLS and MS fulfilled the inclusion criteria. The voxel-wise analysis yielded associations between RLS and MS in the subcortex of the left gyrus precentralis.

**Conclusion:**

Our voxel-wise analysis shows associations in the subcortex of the left gyrus precentralis. Thus, our data suggests that a dysfunction of the efferent motor system due to cerebral lesions may contribute to the pathophysiology of RLS in MS.

## Introduction

Multiple sclerosis (MS) is an inflammatory demyelinating disorder of the central nervous system that is known to be frequently associated with various comorbidities, often adversely affecting the quality of life of the patients and the course or status of the disease [1].

Restless legs syndrome (RLS) is a sleep-related movement disorder characterized by a distressing urge to move the legs and relief by movement, usually accompanied by unpleasant sensory perceptions like pain and paresthesia, predominantly occurring at night [2]. The pathophysiology of RLS is not completely understood; however, there is evidence that RLS may be linked with central iron deficiency and disturbance [3]. While a strong genetic influence in idiopathic RLS is assumed, conditions that cause secondary RLS include iron deficiency, pregnancy, and kidney disease [3].

Prevalence and incidence of RLS is increased in MS, for which reason studies have suggested that MS is a cause of secondary RLS [4, 5]. While RLS is reported to be approximately 7% in general population, its prevalence in MS patients ranges from 12.12 to 57.50% [2, 4]. Furthermore, MS is associated with a fourfold increased odds for RLS.

The pathophysiological background that may lead to MS-related RLS is still under debate. As iron is also an important cofactor in the myelination of the central nervous system, disturbances in the brain iron metabolism have been suggested as possible mechanism [6].

A lot of MS symptoms, especially motor deficits, can be attributed to certain lesions in the brain or the spine. Also, non-motor symptoms, like sensory phenomena and autonomous deficits, have been shown to be attributed to specified cerebral lesions sites [7, 8].

Epidemiological studies found evidence of an association of RLS with the presence of cervical spinal cord lesions [9, 10]. Analyzing the regional MS lesion loads, Manconi et al. [5] demonstrated that the occurrence of MS-related RLS correlated with an increased presence of cervical spinal cord lesions.

However, little is known about the relationship between cerebral, especially supratentorial lesion sites and the development of RLS so far. Previous studies used a dichotomous descriptive statistical analysis or analyzed the lesion load by using a region of interest-based approach [5, 9, 10]. To our knowledge, no neuroimaging study has systematically assessed possible correlations with specific lesion sites using a statistical voxel-wise analysis of the whole brain.

Therefore, in our study, we hypothesized that demyelinating MS lesions in the brain might contribute to RLS in people with MS (pwMS). To determine associations between occurrence of RLS related to MS and cranial MS lesion location, we applied a voxel-based lesion symptom mapping (VLSM) analysis [11–13].

## Materials and methods

### Patients

This study was a retrospective analysis of multiple sclerosis patients seen between 2006 and 2020 at the Department of Neurology of the University Hospital Erlangen (Erlangen, Germany). All patients gave written and informed consent prior to inclusion. After examination, medical reports of pwMS are stored in a digital database. In these records, we performed a search for pwMS and RLS by using the key words “multiple sclerosis” and “restless legs syndrome.” We studied patients who fulfilled the following inclusion criteria: (1) patients with relapsing–remitting, secondary progressive, primary progressive or unclassified MS; (2) aged 18 to 65 years; (3) available medical reports containing details of the medical history. We excluded patients with the following conditions: (1) imaging or MRI sequences of poor quality; (2) structural cerebral diseases other than MS; (3) patients with known iron deficiency or an estimated glomerular filtration rate of lower than 60 ml/min/1.73 m^2^. In our outpatient clinic, relevant parameters (serum ferritin, cut-off < 20 µg/l; serum iron, cut-off < 60 µg/dl) are determined in patients with clinical symptoms of RLS and low hemoglobin levels (cut-off 12 g/dl).

Recent guidelines on the diagnosis of RLS were used [2]. Diagnosis of MS was defined according to the current version of the McDonald criteria [14, 15]. The degree of physical disability was determined using the Expanded Disability Status Scale (EDSS) score [16]. We established a control group of pwMS of the database without documented RLS to compare the imaging characteristics of patients with and without RLS secondary to MS. Control patients were matched for gender and EDSS score. A variability of 0.5 points in EDSS score was tolerated, if no exact match was available [17].

### MRI examination and lesion mapping

In all patients 1.5 or 3 Tesla (T) MRI of the brain was obtained. 3 T was performed in six individuals in the cohort and five in the control group (*p* = 0.745). MRI scans included axial T2-weighted sequences with a slice thickness of 5 mm maximum. MRI T2-weighted sequences were obtained using the following parameters for 1.5 T (flip angle 145°, field of view 100mm, acquisition matrix 0/320/208/0, repetition time 5000 msec, echo time 96 msec) and for 3 T (flip angle 83°, field of view 100mm, aquisition matrix 0/320/320/0, repetition time 5910 msec, echo time 94 msec). In addition, to detect cortically located lesions, axial fluid-attenuated inversion recovery (FLAIR) sequences were acquired. MS lesions were assessed on anonymized T2-weighted MRI sequences by two experienced raters (KF and KW) and lesion boundaries checked by a blinded third investigator (FS). MS lesions were delineated manually with the MRIcron software [18, 19].

Lesions were delineated only on T2-weighted MRI scans, but FLAIR weighted scans were used to control the lesion location for consistency [13, 17]. The MRI scans and lesion shapes were converted into stereotaxic space with the normalization algorithm of SPM12 and the Clinical Toolbox for SPM [20–22]. Finally, the MR images were conferred to the T1 template with a resampled voxel size of 1 × 1 × 1 mm^3^ using the MR-segment-normalize algorithm of the Clinical Toolbox [20].

### Statistical analysis

We determined the lesion overlap, i.e., the overlap of lesioned voxels, of all pwMS with and without restless legs syndrome [12, 13, 17]. Dichotomous overlap values of MS-lesion sites identified in the VLSM analysis were correlated with the dichotomous behavioral variable, that is, the presence or absence of RLS using the Liebermeister test with 4000 permutations [17]. To reduce noise, i.e., the appearance of isolated irrelevant voxels, a threshold was applied. Therefore, only voxels that were lesioned in at least four individuals were included in the analysis. We used a family-wise error (FWE) correction of *p* < 0.05 to control for multiple comparisons. The calculation of lesion volumes of MS lesions was performed with the non-parametric mapping (NPM) software implemented in the MRIcron software package [19]. Affected voxels were overlaid on the Automated Anatomic Labeling (AAL) atlas to determine damaged brain regions. The peak coordinates of the involved regions are demonstrated in the Montreal Neurological Institute (MNI) space.

Demographic and clinical and data were tested for normal distribution using the Shapiro–Wilk test and are presented as median and interquartile ranges. As appropriate, data were compared using the χ^2^ test or Mann–Whitney *U* test. Statistical significance was assumed for *p* < 0.05. For statistical calculations, we used a commercially available statistic program (SPSS 20.0; IBM, Armonk, NY).

## Results

### Patient characteristics

**Fig. 1:**
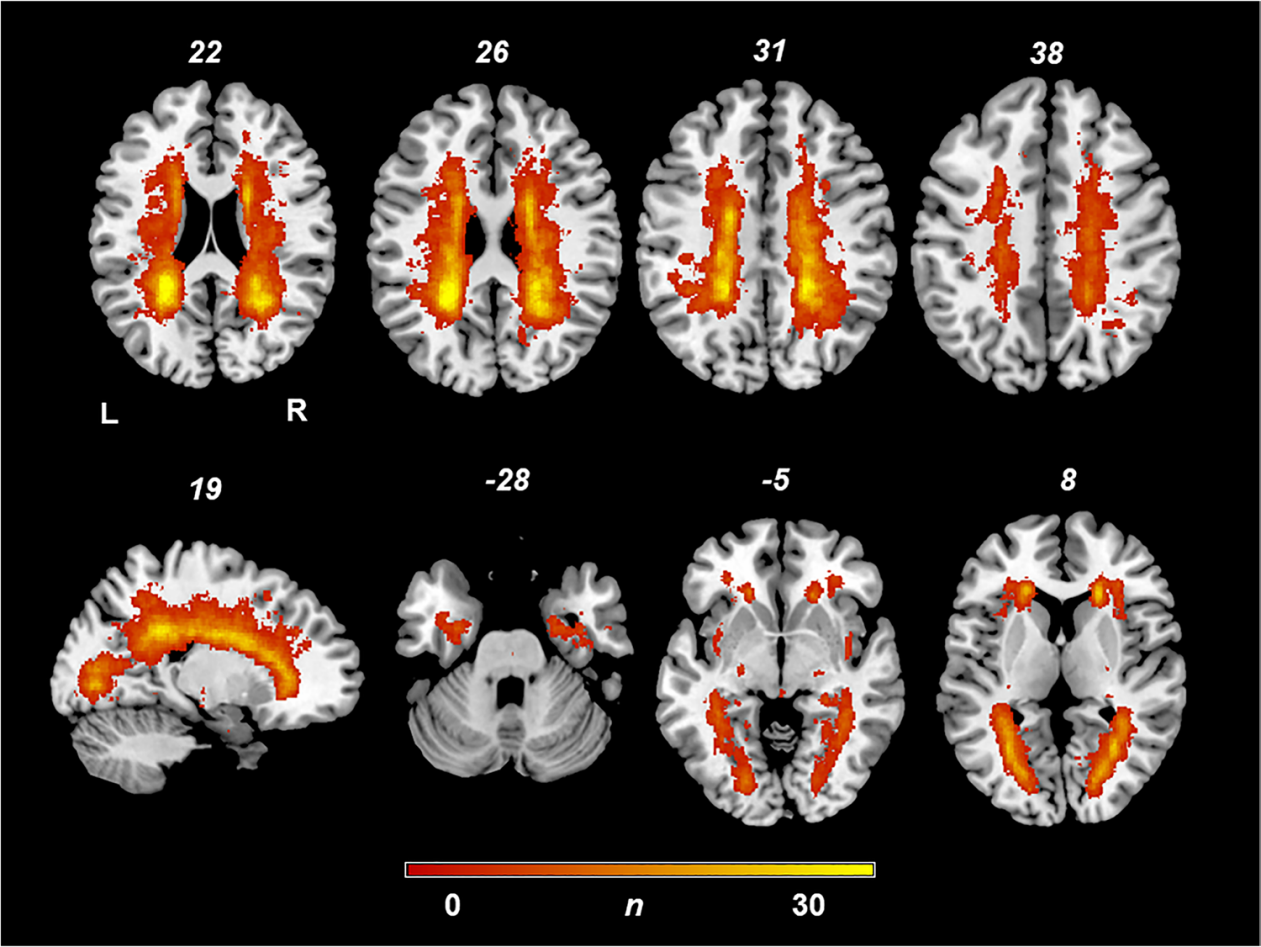
Lesion overlap map in the axial plane of all patients with RLS in MS. Overlap and distribution of lesions in all 40 patients with RLS without a threshold. The number of overlapping lesions is illustrated by different colors, coding increasing frequencies from dark red to yellow. The highest lesion overlap, that is, the highest prevalence of individuals with lesions in a given voxel, was seen in the periventricular regions. L, left hemisphere; n, number of overlaps with a lesion in a given voxel; R, right hemisphere

We identified 40 pwMS with RLS who fulfilled the inclusion criteria. Sixty-six percent of the patients were female and 34% were male. Table 1 demonstrates clinical parameters and imaging characteristics of patients with RLS in multiple sclerosis and of the matched cohort. A total of 74% were classified as relapsing–remitting MS, 16% as secondary progressive MS, and 10% as primary progressive MS at the time of brain imaging. Patients with RLS had a higher patient age, disease duration, and total lesion volume. The EDSS score did not differ between patients with and without RLS. Figure 1 shows the lesion distribution and lesion overlap of all patients with and without RLS. The highest lesion overlap, i.e., the highest prevalence of individuals with lesions in a given voxel, was seen in the periventricular regions.

**Table 1:** Clinical parameters of patients with and without restless legs

	With restless legs (*n* = 40)	No restless legs (*n* = 40)	*P*
Age, years; median (IQR)	48 (54–41)	39 (53–33)	0.035^a^
Disease duration, months; median (IQR)	113 (210–59)	64 (122–15)	0.012^a^
EDSS; median (IQR)	3 (4–2)	2.5 (4–1)	0.149^a^
Female/male	11/29	16/24	0.237^b^
Lesion volume T2, voxels; median (IQR)	11,363 (22,891–6656)	18,738 (39,747–10,114)	0.018^a^
MS type (RRMS/SPMS/PPMS/n.a.)	26/7/6/1	33/6/1/0	0.140^b^

*IQR* interquartile range, *EDSS* Expanded Disability Status Scale, *n.a.* MS type not classified, *RRMS* relapsing–remitting multiple sclerosis, *SPMS* secondary progressive multiple sclerosis

^a^*P* value derived from Mann–Whitney *U* test

^b^*P* value derived from χ^2^ test (two-tailed)

### Voxel-based lesion symptom mapping

The non-parametric voxel-wise analysis using Liebermeister statistics showed associations between RLS and a total of 115 lesioned voxels. Table 2 lists the brain areas with the lesioned voxels resulting from the Liebermeister analysis according to the Automated Anatomical Labeling (AAL) atlas, the corresponding voxel count, and the peak coordinates in MNI space.

**Table 2:** Results from the voxel-wise Liebermeister analysis

Lesion site	Voxels	X	y	Z
Sup coronar radiation (l)	31	− 25	− 5	38
Sup coronar radiation (r)	1	21	− 13	22
Ant coronar radiation (l)	5	− 22	24	22
Genu of corp callosum	2	− 16	27	− 6
Body of corp callosum	2	− 19	− 21	35
Splenium of corp callosum	3	− 18	− 47	23
Post coronar radiation (r)	1	28	− 35	21
Calcarine (r)	4	18	− 77	5

Brain areas according to the areas defined in the Automated Anatomical Labeling (AAL) atlas in which 49 of the 115 lesioned voxels were associated with restless legs in the lesion sites mentioned above, as well as corresponding voxel counts and peak coordinates in MNI space are shown (brainstem and subcortical lesions not included)

*l* left, *r* right

**Fig. 2:**
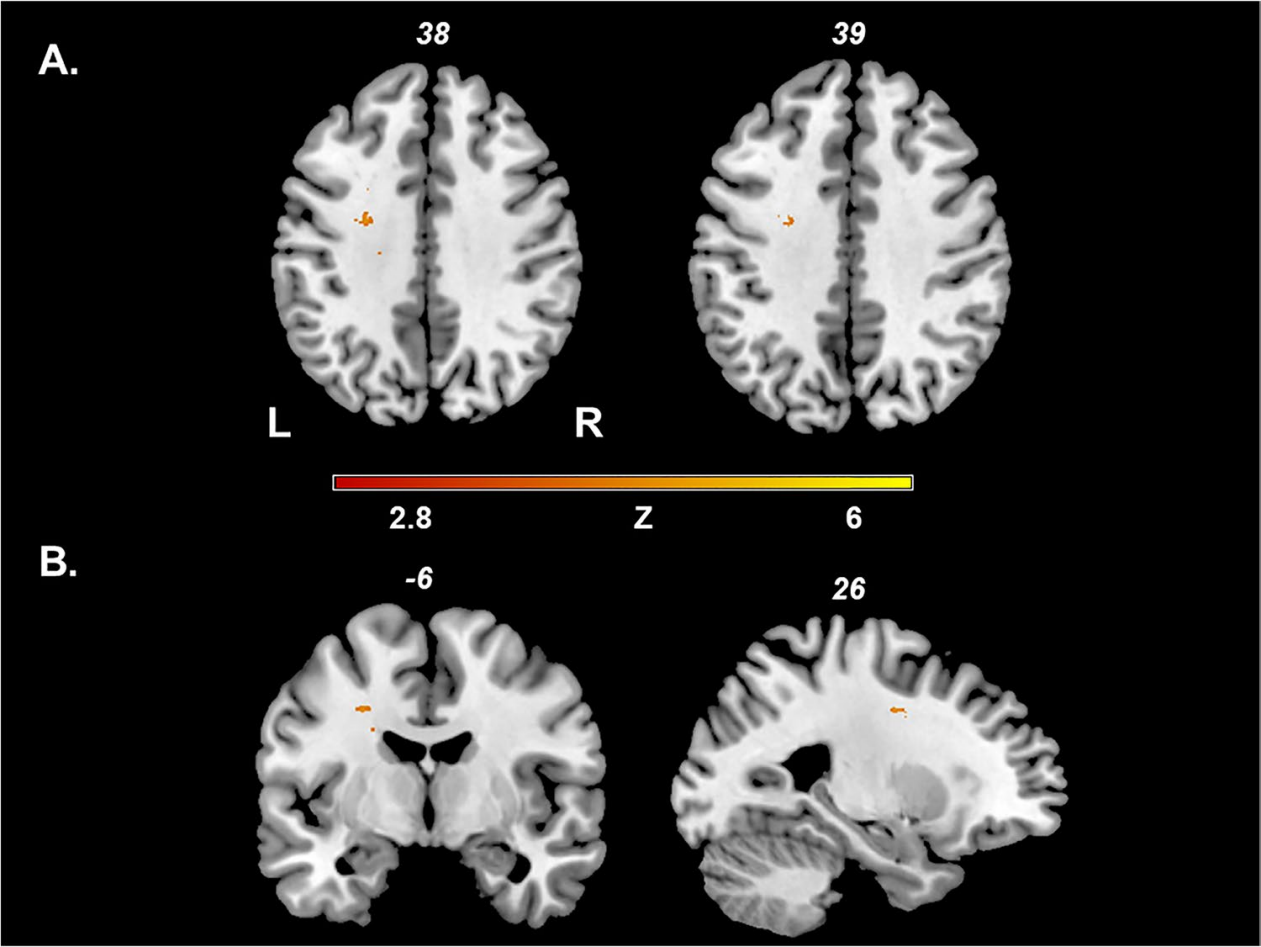
Results of the VLSM analysis. Lesioned voxels depicted in colors from red to yellow remained associated with RLS in pwMS. We conducted non-parametric Liebermeister statistics to assess correlations. Only voxels that were damaged in at least 4 patients were included in the analysis. To control for multiple comparisons, we applied a family‐wise error correction of *P* < 0.05. Restless legs syndrome correlated significantly with a lesion cluster of the subcortex of the left gyrus precentralis. L, left hemisphere; R, right hemisphere; VLSM, voxel‐based lesion‐symptom mapping

Figure 2 demonstrates the results of the Liebermeister test. RLS correlated significantly among others, with brain regions mainly located in frontal white matter and regions of the temporo-occipital cortex.

## Discussion

RLS is known to be associated with MS, having a fourfold increased prevalence in pwMS than in the general population [4]. However, the pathophysiological background for this relationship still remains unclear. Various neurological deficits and phenomena have been shown to be attributed to MS lesions in specific neuroanatomical regions [7, 17]. In pwMS, previous studies found an association of RLS with the presence of cervical MS lesions [5, 10].

However, especially the role and patterns of supratentorial lesions in the pathogenesis of RLS in MS remains unclear.

In the present study, we tried to identify brain regions associated with RLS in pwMS using a voxel-wise-based lesion imaging approach. The VLSM analysis identified specific sites of cerebral MS lesions to be associated with the RLS. The voxel-wise comparison of the absence or presence of RLS between patients with and without lesions in a given voxel (Table 2, Fig. 2) yielded associations between RLS and T2-weighted MS lesions in the white matter close to the cortex in the left hemisphere.

Current concepts of RLS assume a dysfunction of the dopaminergic system, and damages including demyelination along this route may cause a dysfunction manifesting as RLS [4]. In fact, we did not detect lesions in brain regions predominantly involved in the dopaminergic motor control, like the striatum, thalamus, hypothalamus, substantia nigra, or the limbic system.

In our study, we found a lesion cluster associated with RLS in the white matter in proximity of the gyrus precentralis, which may indicate a damage of the corticospinal tract. A decrease of white matter volumes of the precentral gyri has been shown in RLS before [23]. RLS and the periodic limb movements occurring in RLS patients share certain similarities with the Babinski sign, which is pathognomonic for an affection of the corticospinal neurons [4]. Symptomatic dyskinesia in MS has been demonstrated to be associated, among others, with lesions in the central nervous motor system, like the basal ganglia and the pyramid tract [17]. Interestingly, we did not detect any significant lesion cluster in lower structures like the basal ganglia and the infratentorial pyramidal tract, structures of the motor control or the efferent motor system that have been shown to be affected in other sorts of movement disorders in MS [17, 24]. A reason for this may be the predilection of MS lesions for the periventricular white matter, as demonstrated in the lesion overlap (Fig. 1). Furthermore, we did not find any evidence for an involvement of afferent sensory pathways in the pathogenesis of symptomatic RLS in MS [3]. Our study suggests a damage of those infratentorial motor pathways plays a minor role in RLS in pwMS, which could be more a result of disinhibition by a disruption of supratentorial structures.

The mechanisms leading to RLS due to supratentorial, e.g., subcortical, MS lesions remain unclear. Local demyelination may lead to a damage and a disruption of neurological pathways and interconnections, consecutively with an inhibition or disinhibition of secondary neurological structures [25, 26]. As postulated before, spinal locomotor excitability may be caused by impairment of inhibitory supraspinal locomotor descending neurons to the spinal gray matter [24].

Alternatively, demyelination may cause ephaptic activity, ephaptic spread, and hyperexcitability in the affected regions of the central nervous system, as proposed in paroxysmal dyskinesia in MS before [17, 25]. In fact, demyelination is known to render axons hypersensitive [27].

Notably, our detected lesion cluster was located in the left hemisphere, although patients did not report any lateralization of the RLS complaints in the study. The reason for this remains speculative. However, lesion lateralization has been reported in patients with bilateral pyramidal and extrapyramidal symptoms in MS before [7, 17]. Also in RLS patients without MS, white matter abnormalities in areas of the efferent motor system and sensorimotor integration, lateralized to the left hemisphere, have been found using diffusion-tensor imaging [28]. As a hypothesis, RLS in pwMS may occur due to a disinhibition because of distinct lesions in the lateralized central motor control or motor pathways in a dominant (left) hemisphere. Consequently, supratentorial lesions may only partially account for RLS in pwMS.

Subjects with symptomatic RLS had a higher disease duration, a factor that might increase the probability of a MS lesion localized in the efferent motor system that may lead to symptomatic RLS. Several studies have demonstrated a correlation between MS severity [5] and the occurrence of symptomatic RLS [3, 10]. However, the cerebral lesion load was lower in individuals with RLS in our cohort. Therefore, the previously reported higher clinical MS severity in RLS patients may possibly have resulted of a higher rate of lesions of the spinal cord.

In summary, in this study, we were able to detect a lesion cluster in the left white matter next to the gyrus precentralis associated with RLS in pwMS. As hypothesis, pwMS with symptomatic RLS may partially suffer from a dysfunction of the corticospinal tract due to MS lesions.

## Limitations

Several limitations of this study need to be considered. The manifestations of MS are very heterogeneous. Because of the retrospective nature, the diagnosis of RLS based on patients’ records was not systematically assessed, which may have led to underreporting. Although patients were asked carefully, we cannot rule out that patients of the control group had no RLS. An exact classification of the type of RLS, although desirable, could not be realized. Therefore, other causes for RLS than MS were not definitely excluded. A more well-defined cohort of RLS due to MS or a higher sample size may have produced more clear results. MS disease duration was higher in pwMS with RLS than in the control group. As disease duration is associated with RLS severity, we cannot exclude that this may have affected our results. The higher age in the cohort is a confounding factor, too. Additional matching for age and disease duration was not realizable, so we cannot exclude that differences in these parameters between both groups may have biased our results.

We did not have a standardized MR protocol, which could have influenced the results. Although MRI slice thickness of 5 mm meets the criteria of the current guidelines for MS, a lower thickness would have been desirable [14, 15]. Spinal lesions are known to cause restless legs syndrome in MS. Unfortunately, the VLSM approach is not validated for correlating spinal lesions. Methodically, this study only was able to examine cerebral lesions, which could have biased the results. However, it seems unlikely that the presence or absence of spinal cord lesions may have produced false-positive results, i.e., the cerebral lesion cluster in the Liebermeister analysis of the brain. Tissue damage in MS may be diffused and not detectable in MRI, but also impair neurons involved in the development of RLS in MS.

## Conclusion

In conclusion, this study was able to identify a lesion cluster in the subcortex close to the left gyrus precentralis significantly associated with RLS in pwMS. Thus, our data suggests that a dysfunction of the efferent motor system due to cerebral lesions may contribute to the pathophysiology of RLS in MS. Further information about the functional alteration of lesioned neuronal networks and connectivity is needed and should be the object of future studies.

## Data Availability

Available on request.
